# Penetrating Neck Injury to the Superior Thoracic Artery Managed by Video-Assisted Thoracoscopic Surgery

**DOI:** 10.1155/2013/413462

**Published:** 2013-02-07

**Authors:** Victor W. Wong, Stephanie D. Gordy, Martin Schreiber, Brandon H. Tieu

**Affiliations:** ^1^Department of Surgery, Oregon Health and Science University, 3181 SW Sam Jackson Park Road, Portland, OR 97239, USA; ^2^Division of Cardiothoracic Surgery, Department of Surgery, Oregon Health and Science University, 3181 SW Sam Jackson Park Road, L353, Portland, OR 97239, USA

## Abstract

Penetrating trauma to the axillary artery and its branches is uncommon and associated with high morbidity and mortality. Open exploration is mandated in hemodynamically unstable patients, but surgical exposure can be difficult due to the concentration of vital structures and complex anatomy in this region. Computed tomographic angiography is a potential diagnostic modality in hemodynamically stable patients. In these patients, endovascular therapies may provide a feasible means of controlling hemorrhage while minimizing surgical complications. A high incidence of concomitant intrathoracic injury has resulted in an expanding role for video-assisted thoracoscopic surgery. In this paper, we present a case of penetrating injury to the superior thoracic artery that was not amenable to endovascular therapy and was ultimately managed with thoracoscopic surgery.

## 1. Introduction

The subclavian and axillary arteries, involved in about 3% of penetrating neck and chest trauma and <5% of all vascular trauma, are relatively protected by overlying musculoskeletal structures [1, 2]. However, when injury does occur, morbidity and mortality can be substantial due to the density of vital surrounding structures and the difficulty in both detecting and controlling hemorrhage [3, 4]. Interventional radiology and endovascular strategies may be a viable option for select upper extremity arterial injuries in stable patients [5, 6]. Open surgical approaches to the subclavian or axillary artery are indicated in the hemodynamically unstable patient but associated with significant morbidity. More recently, video-assisted thoracoscopic surgery (VATS) has been increasingly utilized for the diagnosis and treatment of penetrating injuries to the torso in the acute trauma setting [7, 8]. 

## 2. Case Report

**Figure 1:**
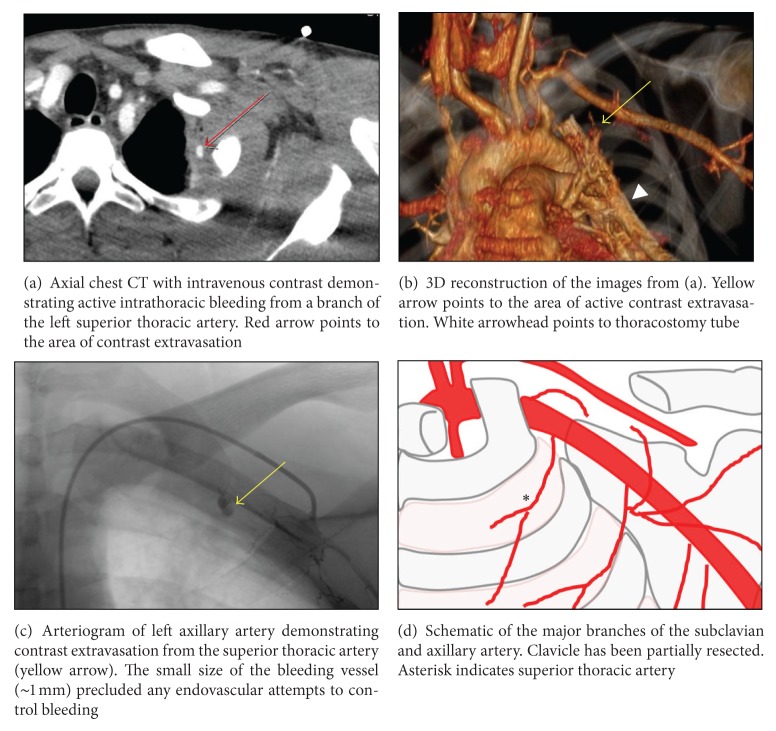
Radiographic images and schematic of vascular injury.

A 28-year-old healthy man was stabbed in the neck above the left clavicle with a long knife. He was brought to the hospital in stable condition with an oxygen saturation of 100%. He was mildly tachypneic (22 breaths/min) and complained of left-sided chest pain. Exam revealed a 2 cm supraclavicular wound without evidence of external bleeding or expanding hematoma, a strong left radial pulse, and no motor sensory deficits. A portable chest radiograph revealed a moderate-sized left pneumothorax. A tube thoracostomy was performed in the emergency department and immediately yielded 200 mL of blood. Chest computed tomographic angiography and neck computed tomographic angiography (CTA) revealed a hematoma posterior to the clavicle and a small contrast blush from a superior thoracic artery branch into the thoracic cavity (Figures 1(a) and 1(b)). Chest tube output at that time remained minimal, and the patient was hemodynamically stable and was transferred to the surgical ward. 

**Figure 2:**
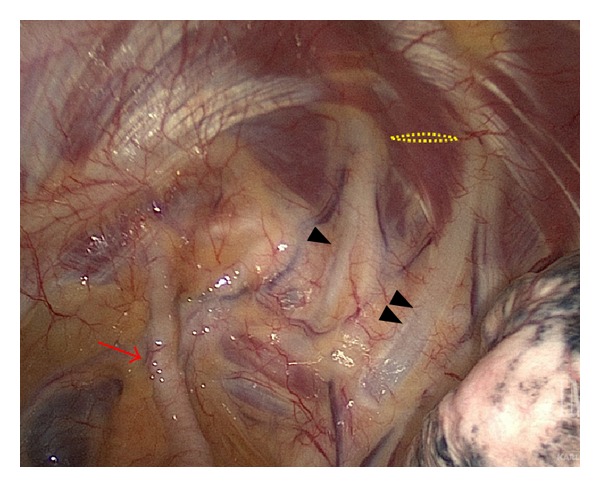
Thoracoscopic view in another patient showing the location of injury. Representative thoracoscopic view of left thoracic inlet demonstrating the left subclavian artery (red arrow), first (black arrowhead) and second (double arrowheads) ribs, and the area of injury (yellow dotted line). The deflated lung is seen in the right lower quadrant. Superior = top.

Chest CT also identified an intrafissural chest tube that was subsequently pulled back two centimeters, resulting in more than one liter of blood over the next hour. The patient was transiently tachycardic (130 beats/min) and hypotensive (90 mm Hg systolic BP) but responded to a bolus of intravenous crystalloid en route to transfer to the intensive care unit. He received 2 units of packed red blood cells for ongoing bleeding and a decrease in hemoglobin to 10.1 g/dL from 11.8 g/dL. The chest tube output decreased to 55 mL of blood over the next hour, and he remained hemodynamically stable. Angiography revealed active bleeding from a branch of the superior thoracic artery that was not amenable to endovascular therapy due to the small (~1 mm) vessel size (Figures 1(c) and 1(d)). Thoracic surgery was consulted and performed a left VATS with evacuation of more than one liter of hematoma and thoracoscopic control of extrapleural bleeding (Endo: Clips Covidien, Mansfield, MA, USA; Surgicel: Ethicon, San Antonio, TX, USA; Gelfoam: Pfizer, New York, NY, USA). A 3 cm defect in the parietal pleura was identified between the first and second ribs (Figure 2). The patient was discharged home on postoperative day five without any chest tubes or complications. 

## 3. Discussion

Penetrating trauma to the neck and chest involves the subclavian or axillary vasculature in about 3% of cases [4, 9]. Although concomitant first rib or clavicle injury may occur in up to 14% of cases, significant morbidity is associated with brachial plexus injury [4, 10–13]. Up to 28% of patients will also have intrathoracic injuries, and the presence of both venous and arterial injuries is associated with significant mortality [4]. Most victims will die before reaching the hospital or reach the hospital in extremis [3]. “Hard” signs of vascular injury that mandate emergent open surgical treatment include severe bleeding, unexplained hypotension, expanding hematoma, or absent peripheral pulses. “Soft” signs of vascular injury include stable small hematomas, minor continuous bleeding, or mild hypotension. 

In situations where immediate surgical exploration is not indicated, diagnostic imaging may be warranted to optimize the treatment approach. Plain radiographs may be used to diagnose the presence of pneumothorax or hemothorax and can identify foreign bodies. Doppler ultrasound has been utilized in some centers as a noninvasive tool to effectively detect vascular injury but is user dependent, and expertise may not be widely available [1, 4]. Routine arteriography was traditionally advocated for all penetrating injuries to zone 1 of the neck (between clavicles and cricoid cartilage), but recent studies have questioned the low yield and the invasive nature of this approach [14, 15]. Multidetector CT angiography has been shown to be highly sensitive and specific for evaluating penetrating neck and chest trauma and has been recommended as the first-line approach for stable patients with suspected vascular trauma [16, 17].

Up to 50% of penetrating injuries to the subclavian or axillary artery may be amenable to endovascular therapy [5]. Endovascular repair has been associated with shorter operative times, less blood loss, and comparable short-term patency compared to open repair [6, 18, 19]. However, longterm outcome studies after endovascular intervention are lacking. Open surgical repair is indicated for unstable patients, but exposure of injured vessels is often challenging. In general, infraclavicular incisions with either retraction or resection of the medial clavicle can provide adequate exposure to the subclavian artery. Division of the pectoral muscles can provide exposure to the axillary artery. The addition of a median sternotomy and anterolateral thoracotomy exposes the proximal subclavian vessels via a “trapdoor” approach. 

In the acute trauma setting, VATS has traditionally been used for retained hemothorax or to diagnose diaphragmatic injury [20]. A recent prospective multicenter study found VATS to be the most commonly performed intervention following tube thoracostomy for retained hemothorax (33.5%) with a success rate of 70% [21]. Other studies have demonstrated its utility for chest wall bleeding, diagnosing transmediastinal or cardiac injury, persistent pneumothorax, and blunt chest injury [22, 23]. Despite its increasing range of indications, VATS should only be considered in the hemodynamically stable trauma patient. In summary, we present a case of penetrating injury to the thoracic inlet with ongoing intrathoracic bleeding from a superior thoracic artery branch that was not amenable to endovascular repair but successfully managed with thoracoscopic surgery.
